# Supplementary material for: E-Volve: understanding the impact of mutations in SARS-CoV-2 variants spike protein on antibodies and ACE2 affinity through patterns of chemical interactions at protein interfaces
Source: PeerJ. 2022 Mar 22;10:e13099. doi: 10.7717/peerj.13099 (PMC8953562; doi:10.7717/peerj.13099)
Supplement: Supplemental Information 1 — Figure S1. Inter-chain interactions in spike protein for Delta (PDB ID 7V8B) and Omicron (PDB ID 7T9K) variants. Figure S2. MD results for the RBD and RBM motifs. Figure S3. Influence of the differential context of the loop α1′/β1′ on the complementarity of the WT and P1 RBDs at the receptor and antibodies. Table S1. List of 3D-structures (PDB IDs) collected from Protein Data Bank (https://www.rcsb.org/). Data was collected on April 30, 2021. Table S2. RMSD concerning the starting structure, packing and mobility parameters recovered at the respective MD simulations for the WT and P1 RBD. [file peerj-10-13099-s001.pdf]

Supplementary Material

1 Supplementary Figures and Tables

1.1 Supplementary Figures

Figure S1. Inter-chain interactions in spike protein for Delta (PDB ID 7V8B) and Omicron (PDB ID 7T9K) variants.

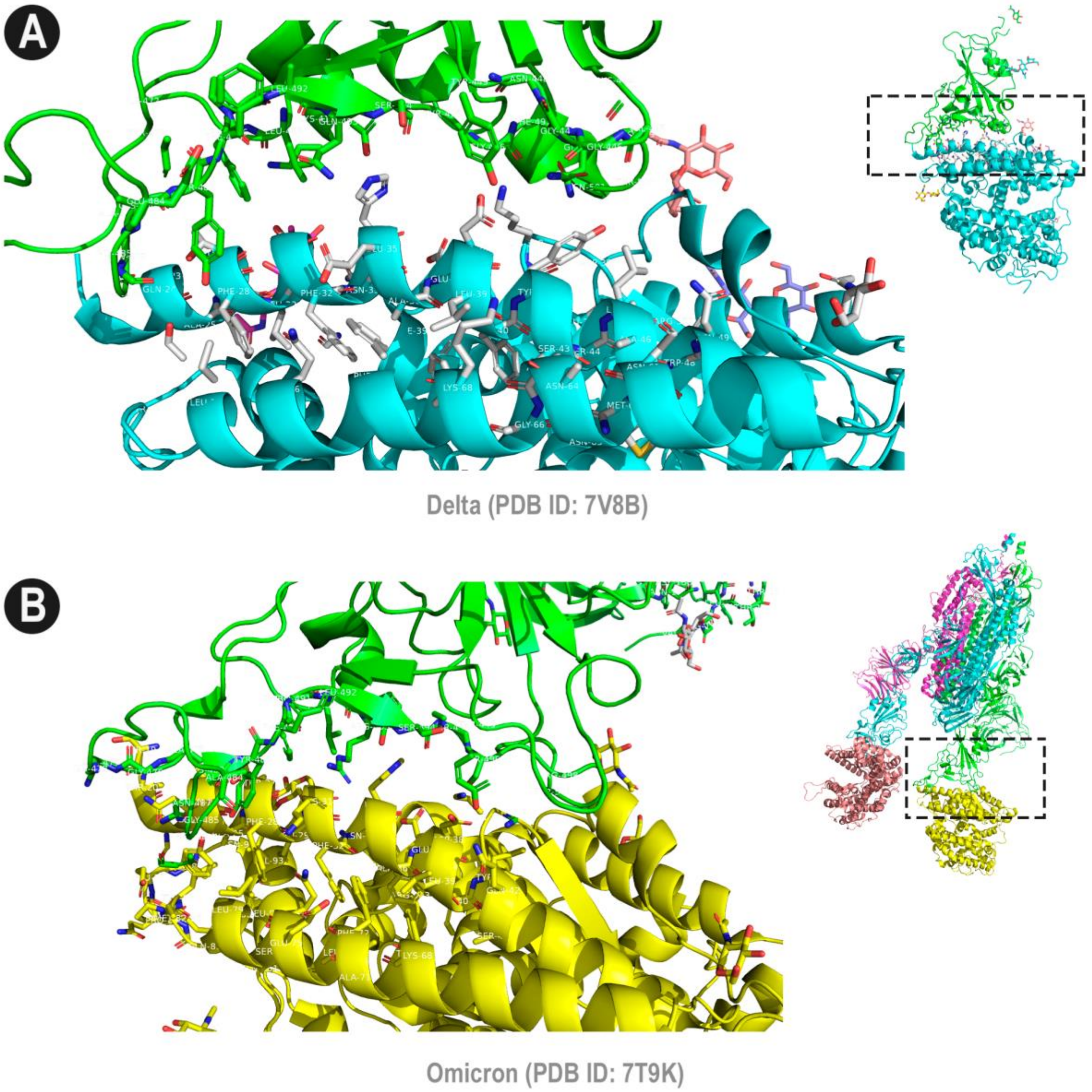

Figure S2. MD results for the RBD and RBM motifs.

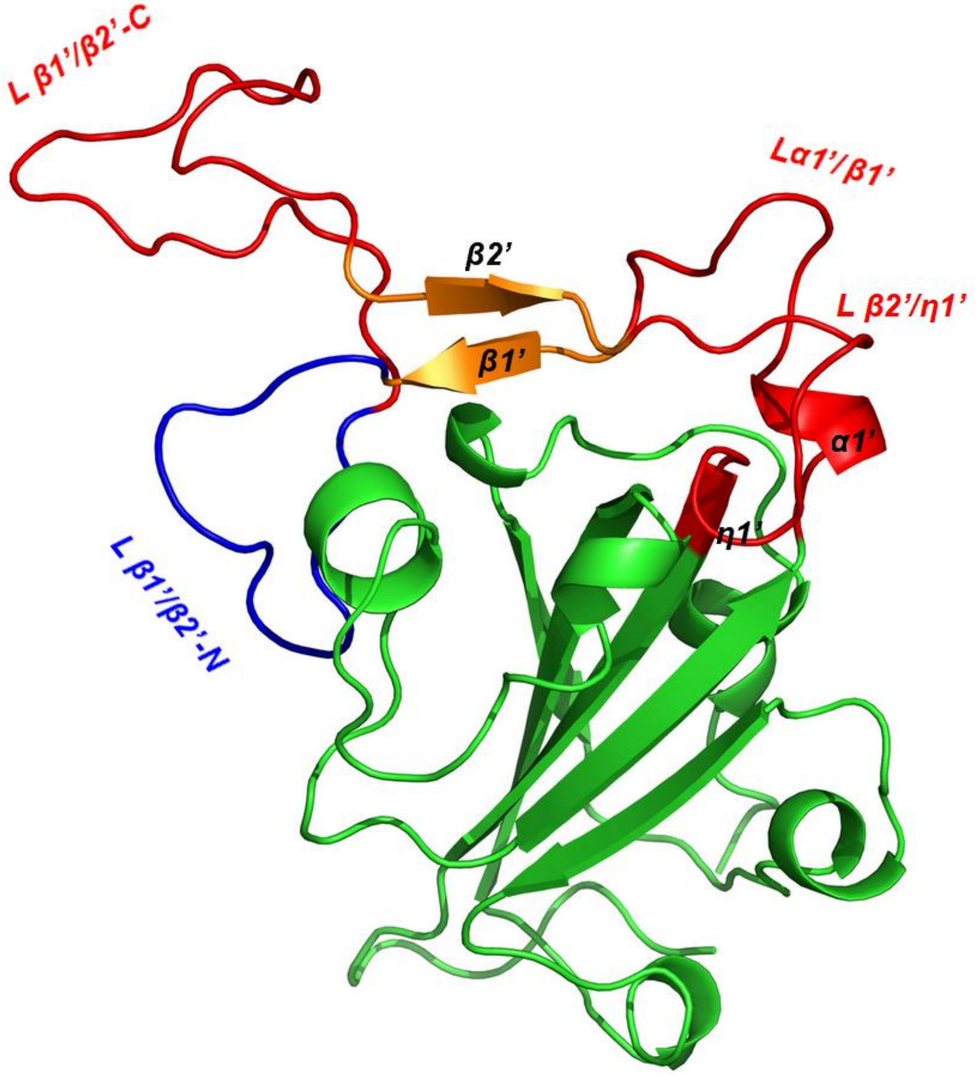

Figure S3 - Influence of the differential context of the loop α1'/β1' on the complementarity of the WT and P1 RBDs at the receptor and antibodies.

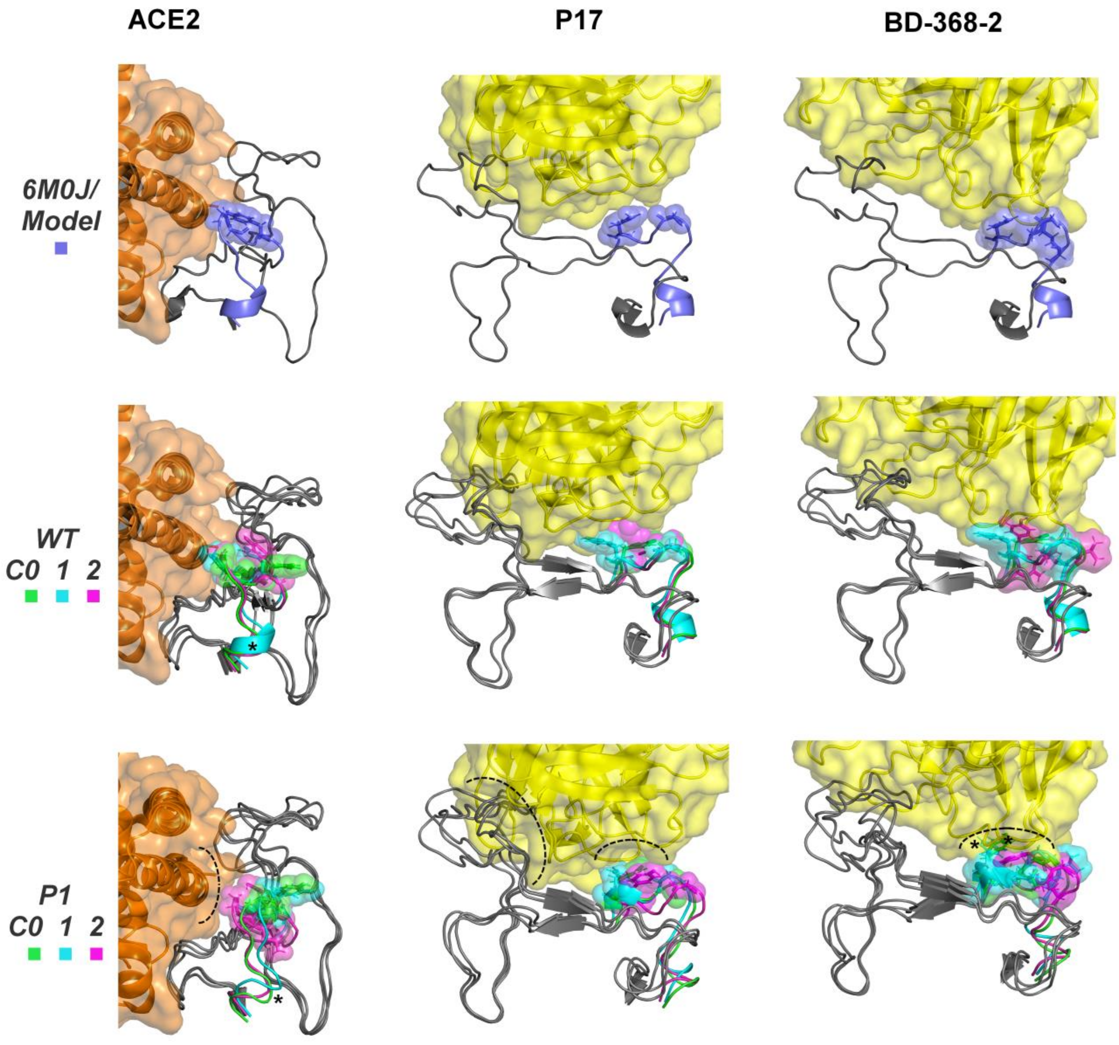

From the top to the bottom rows, the structure of the complex at the crystallographic structure (6M0J, being this also the same interface recovered for the P1 model using the MODELLER procedure), the superposition of the cluster centroids C0, C1 and C2 from the WT's MD simulations at each protein and the superposition of the respective cluster centroids from the P1's MDs are depicted. From the left to the right, the complexes with ACE2 (ACE2 structure from PDB:6M0J), P17 (antibody structure from PDB:7CWN and BD-368-2 (antibody structure from PDB:7CHH) are shown. The residues from loop α1'/β1' (438-450) are colored in purple (6M0J/Model interface), green (C0), cyan (C1) and magenta (C2). The key residues for interaction of this segment with the receptor and antibodies (G446, G447 and Y449) are shown in sticks and as transparent vdw spheres. The asterisks highlight special challenges to be overcome at the P1 loop α1'/β1' binding: the higher disorder of the helix α1' carrying higher mobility; the steric hindrance between the Y449 and the BD-368-2 interface at the expanded loop on the C0 and C1 structures. The dashed arc in P1:ACE2 complex highlights the H1 helix protuberance over the core region of RBM; in P1:P17 depicts the major sites of contact with RBM, loops β1'/β2' (majorly) and α1'/ β1'; in P1:BD-368-2 highlights the higher displacement of the binding interface for loop α1'/β1'.

1.2 Supplementary Tables

Table S1. List of 3D-structures (PDB IDs) collected from Protein Data Bank (https://www.rcsb.org/). Data was collected on April 30, 2021.

PDB ID	Description
6m0j	Crystal structure of SARS-CoV-2 spike receptor-binding domain bound with ACE2
6w41	Crystal structure of SARS-CoV-2 receptor binding domain in complex with human antibody CR3022
6w7y	Crystal structure of SARS-CoV and SARS-CoV-2 reactive human antibody CR3022
6xc2	Crystal structure of SARS-CoV-2 receptor binding domain in complex with neutralizing antibody CC12.1
6xc3	Crystal structure of SARS-CoV-2 receptor binding domain in complex with antibodies CC12.1 and CR3022
6xc4	Crystal structure of SARS-CoV-2 receptor binding domain in complex with neutralizing antibody CC12.3
6xc7	Crystal structure of SARS-CoV-2 receptor binding domain in complex with antibodies CC12.3 and CR3022
6xcm	Structure of the SARS-CoV-2 spike glycoprotein in complex with the C105 neutralizing antibody Fab fragment (state 1)
6xcn	Structure of the SARS-CoV-2 spike glycoprotein in complex with the C105 neutralizing antibody Fab fragment (state 2)
6xdg	Complex of SARS-CoV-2 receptor binding domain with the Fab fragments of two neutralizing antibodies
6xe1	Structure of SARS-CoV-2 spike protein receptor binding domain in complex with a potent neutralizing antibody, CV30 Fab
6xkp	Crystal structure of SARS-CoV-2 receptor binding domain in complex with neutralizing antibody CV07-270
6xkq	Crystal structure of SARS-CoV-2 receptor binding domain in complex with neutralizing antibody CV07-250

PDB ID	Description
6zdg	Association of three complexes of largely structurally disordered Spike ectodomain with bound EY6A Fab
6zdh	SARS-CoV-2 Spike glycoprotein in complex with a neutralizing antibody EY6A Fab
6zer	Crystal structure of receptor binding domain of SARS-CoV-2 Spike glycoprotein in complex with EY6A Fab
6zfo	Association of two complexes of largely structurally disordered Spike ectodomain with bound EY6A Fab
7a29	Cryo-EM structure of the SARS-CoV-2 spike protein bound to neutralizing sybodies (Sb23) 2-up conformation
7a5r	Complex of SARS-CoV-2 spike and CR3022 Fab (Non-Uniform Refinement)
7a5s	Complex of SARS-CoV-2 spike and CR3022 Fab (Homogeneous Refinement)
7b3o	Crystal structure of the SARS-CoV-2 RBD in complex with STE90-C11 Fab
7beh	Crystal structure of the receptor binding domain of SARS-CoV-2 Spike glycoprotein in complex with COVOX-316 Fab
7bei	Crystal structure of the receptor binding domain of SARS-CoV-2 Spike glycoprotein in complex with COVOX-150 Fab
7bej	Crystal structure of the receptor binding domain of SARS-CoV-2 Spike glycoprotein in complex with COVOX-158 Fab (crystal form 1)
7bek	Crystal structure of the receptor binding domain of SARS-CoV-2 Spike glycoprotein in complex with COVOX-158 Fab (crystal form 2)
7bel	Crystal structure of the receptor binding domain of SARS-CoV-2 Spike glycoprotein in a ternary complex with COVOX-88 and COVOX-45 Fabs
7bem	Crystal structure of the receptor binding domain of SARS-CoV-2 Spike glycoprotein in complex with COVOX-269 scFv
7ben	Crystal structure of the receptor binding domain of SARS-CoV-2 Spike glycoprotein in a ternary complex with COVOX-253 and COVOX-75 Fabs

Supplementary Material

PDB ID	Description
7beo	Crystal structure of the receptor binding domain of SARS-CoV-2 Spike glycoprotein in a ternary complex with COVOX-253H55L and COVOX-75 Fabs
7bep	Crystal structure of the receptor binding domain of SARS-CoV-2 Spike glycoprotein in a ternary complex with COVOX-384 and S309 Fabs
7bwj	crystal structure of SARS-CoV-2 antibody with RBD
7byr	BD23-Fab in complex with the S ectodomain trimer
7c01	Molecular basis for a potent human neutralizing antibody targeting SARS-CoV-2 RBD
7c2l	S protein of SARS-CoV-2 in complex bound with 4A8
7c8v	Structure of sybody SR4 in complex with the SARS-CoV-2 S Receptor Binding domain (RBD)
7c8w	Structure of sybody MR17 in complex with the SARS-CoV-2 S receptor-binding domain (RBD)
7cac	SARS-CoV-2 S trimer with one RBD in the open state and complexed with one H014 Fab.
7cah	The interface of H014 Fab binds to SARS-CoV-2 S
7cai	SARS-CoV-2 S trimer with two RBDs in the open state and complexed with two H014 Fab
7cak	SARS-CoV-2 S trimer with three RBD in the open state and complexed with three H014 Fab
7can	Structure of sybody MR17-K99Y in complex with the SARS-CoV-2 S Receptor-binding domain (RBD)
7cdi	Crystal structure of SARS-CoV-2 antibody P2C-1F11 with RBD
7cdj	Crystal structure of SARS-CoV-2 antibody P2C-1A3 with RBD
7ch4	Crystal structure of the SARS-CoV-2 S RBD in complex with BD-604 Fab

PDB ID	Description
7ch5	Crystal structure of the SARS-CoV-2 S RBD in complex with BD-629 Fab
7chb	Crystal structure of the SARS-CoV-2 RBD in complex with BD-236 Fab
7chc	Crystal structure of the SARS-CoV-2 S RBD in complex with BD-629 Fab and BD-368-2 Fab
7che	Crystal structure of the SARS-CoV-2 RBD in complex with BD-236 Fab and BD-368-2 Fab
7chf	Crystal structure of the SARS-CoV-2 RBD in complex with BD-604 Fab and BD-368-2 Fab
7chh	Cryo-EM structure of the SARS-CoV-2 S-6P in complex with BD-368-2 Fabs
7cjf	Crystal structure of SARS-CoV-2 RBD in complex with a neutralizing antibody Fab
7cr5	Complex structure of a human monoclonal antibody with SARS-CoV-2 nucleocapsid protein NTD
7cwl	SARS-CoV-2 spike protein and P17 fab complex with one RBD in close state
7cwm	Complex of SARS-CoV-2 spike protein and Fab P17 with one RBD in open state and two RBD in closed state
7cwn	P17-H014 Fab cocktail in complex with SARS-CoV-2 spike protein
7cwo	SARS-CoV-2 spike protein RBD and P17 fab complex
7cws	SARS-CoV-2 Spike Proteins Trimer in Complex with FC05 and H014 Fabs Cocktail
7cwu	SARS-CoV-2 spike proteins trimer in complex with P17 and FC05 Fabs cocktail
7czp	S protein of SARS-CoV-2 in complex bound with P2B-1A1
7czq	S protein of SARS-CoV-2 in complex bound with P2B-1A10

Supplementary Material

PDB ID	Description
7czr	S protein of SARS-CoV-2 in complex bound with P5A-1B8_2B
7czs	S protein of SARS-CoV-2 in complex bound with P5A-1B8_3B
7czt	S protein of SARS-CoV-2 in complex bound with P5A-2G9
7czu	S protein of SARS-CoV-2 in complex bound with P5A-1B6_2B
7czv	S protein of SARS-CoV-2 in complex bound with P5A-1B6_3B
7czw	S protein of SARS-CoV-2 in complex bound with P5A-2G7
7czx	S protein of SARS-CoV-2 in complex bound with P5A-1B9
7czy	S protein of SARS-CoV-2 in complex bound with P5A-2F11_2B
7czz	S protein of SARS-CoV-2 in complex bound with P5A-2F11_3B
7d00	S protein of SARS-CoV-2 in complex bound with FabP5A-1B8
7d03	S protein of SARS-CoV-2 in complex bound with FabP5A-2G7
7d0b	S protein of SARS-CoV-2 in complex bound with P5A-3C12_1B
7d0c	S protein of SARS-CoV-2 in complex bound with P5A-3A1
7d0d	S protein of SARS-CoV-2 in complex bound with P5A-3C12_2B
7d4g	A proof of concept for neutralizing antibody-guided vaccine design against SARS-CoV-2
7deo	Crystal structure of SARS-CoV-2 RBD in complex with a neutralizing antibody scFv
7dpm	Crystal structure of SARS-CoV-2 Spike RBD in complex with MW06 Fab

PDB ID	Description
7dx4	The structure of FC08 Fab-hA.CE2-RBD complex
7.00E+23	SARS-CoV-2 spike in complex with the CA521 neutralizing antibody Fab (focused refinement on Fab-RBD)
7eam	immune complex of SARS-CoV-2 RBD and cross-neutralizing antibody 7D6
7jmo	Crystal structure of SARS-CoV-2 receptor binding domain in complex with neutralizing antibody COVA2-04
7jmp	Crystal structure of SARS-CoV-2 receptor binding domain in complex with neutralizing antibody COVA2-39
7jmw	Crystal structure of SARS-CoV-2 spike protein receptor-binding domain in complex with cross-neutralizing antibody COVA1-16 Fab
7jv2	SARS-CoV-2 spike in complex with the S2H13 neutralizing antibody Fab fragment (local refinement of the receptor-binding motif and Fab variable domains)
7jv4	SARS-CoV-2 spike in complex with the S2H13 neutralizing antibody (one RBD open)
7jv6	SARS-CoV-2 spike in complex with the S2H13 neutralizing antibody (closed conformation)
7jva	SARS-CoV-2 spike in complex with the S2A4 neutralizing antibody Fab fragment (local refinement of the receptor-binding domain and Fab variable domains)
7jvc	SARS-CoV-2 spike in complex with the S2A4 neutralizing antibody Fab fragment
7jw0	SARS-CoV-2 spike in complex with the S304 neutralizing antibody Fab fragment
7jwb	SARS CoV2 Spike ectodomain with engineered trimerized VH binder
7k43	SARS-CoV-2 spike in complex with the S2M11 neutralizing antibody Fab fragment
7k45	SARS-CoV-2 spike in complex with the S2E12 neutralizing antibody Fab fragment (local refinement of the RBD and Fab variable domains)
7k4n	SARS-CoV-2 spike in complex with the S2E12 neutralizing antibody Fab fragment

Supplementary Material

**PDB ID**	**Description**
7k8m	Structure of the SARS-CoV-2 receptor binding domain in complex with the human neutralizing antibody Fab fragment, C102
7k8s	Structure of the SARS-CoV-2 S 2P trimer in complex with the human neutralizing antibody Fab fragment, C002 (state 1)
7k8t	Structure of the SARS-CoV-2 S 6P trimer in complex with the human neutralizing antibody Fab fragment, C002 (State 2)
7k8u	Structure of the SARS-CoV-2 S 6P trimer in complex with the human neutralizing antibody Fab fragment, C104
7k8v	Structure of the SARS-CoV-2 S 2P trimer in complex with the human neutralizing antibody Fab fragment, C110
7k8w	Structure of the SARS-CoV-2 S 2P trimer in complex with the human neutralizing antibody Fab fragment, C119
7k8x	Structure of the SARS-CoV-2 S 2P trimer in complex with the human neutralizing antibody Fab fragment, C121 (State 1)
7k8y	Structure of the SARS-CoV-2 S 2P trimer in complex with the human neutralizing antibody Fab fragment, C121 (State 2)
7k8z	Structure of the SARS-CoV-2 S 2P trimer in complex with the human neutralizing antibody Fab fragment, C135
7k90	Structure of the SARS-CoV-2 S 6P trimer in complex with the human neutralizing antibody Fab fragment, C144
7k9z	Crystal structure of SARS-CoV-2 receptor binding domain in complex with the Fab fragments of neutralizing antibodies 298 and 52
7kfv	Structural basis for a germline-biased antibody response to SARS-CoV-2 (RBD:C1A-B12 Fab)
7kfw	Structural basis for a germline-biased antibody response to SARS-CoV-2 (RBD:C1A-B3 Fab)
7kfx	Structural basis for a germline-biased antibody response to SARS-CoV-2 (RBD:C1A-C2 Fab)

PDB ID	Description
7kfy	Structural basis for a germline-biased antibody response to SARS-CoV-2 (RBD:C1A-F10 Fab)
7kkl	SARS-CoV-2 Spike in complex with neutralizing nanobody mNb6
7klg	SARS-CoV-2 RBD in complex with Fab 15033
7klh	SARS-CoV-2 RBD in complex with Fab 15033-7
7kmg	LY-CoV555 neutralizing antibody against SARS-CoV-2
7kmh	LY-CoV488 neutralizing antibody against SARS-CoV-2
7kmi	LY-CoV481 neutralizing antibody against SARS-CoV-2
7kmk	cryo-EM structure of SARS-CoV-2 spike in complex with Fab 15033-7, two RBDs bound
7kml	cryo-EM structure of SARS-CoV-2 spike in complex with Fab 15033-7, three RBDs bound
7kn5	Crystal structure of SARS-CoV-2 receptor binding domain complexed with nanobodies VHH E and U
7kn6	Crystal structure of SARS-CoV-2 receptor binding domain complexed with nanobody VHH V and antibody Fab CC12.3
7kn7	Crystal structure of SARS-CoV-2 receptor binding domain complexed with nanobody VHH W and antibody Fab CC12.3
7ks9	Cryo-EM structure of prefusion SARS-CoV-2 spike glycoprotein in complex with 910-30 Fab
7kxj	SARS-CoV-2 spike protein in complex with Fab 15033-7, 3-"up", asymmetric
7kxk	SARS-CoV-2 spike protein in complex with Fab 15033-7, 2-"up"-1-"down" conformation
7kzb	Potent SARS-CoV-2 binding and neutralization through maturation of iconic SARS-CoV-1antibodies

Supplementary Material

PDB ID	Description
7l02	Cryo-EM structure of SARS-CoV-2 2P S ectodomain bound to one copy of domain-swapped antibody 2G12
7l06	Cryo-EM structure of SARS-CoV-2 2P S ectodomain bound to two copies of domain-swapped antibody 2G12
7l09	Cryo-EM structure of SARS-CoV-2 2P S ectodomain bound domain-swapped antibody 2G12 from masked 3D refinement
7l0n	Circulating SARS-CoV-2 spike N439K variants maintain fitness while evading antibody-mediated immunity
7l2c	Crystallographic structure of neutralizing antibody 2-51 in complex with SARS-CoV-2 spike N-terminal domain (NTD)
7l2d	Cryo-EM structure of NTD-directed neutralizing antibody 1-87 in complex with prefusion SARS-CoV-2 spike glycoprotein
7l2e	Cryo-EM structure of NTD-directed neutralizing antibody 4-18 in complex with prefusion SARS-CoV-2 spike glycoprotein
7l2f	Cryo-EM structure of NTD-directed neutralizing antibody 5-24 in complex with prefusion SARS-CoV-2 spike glycoprotein
7l3n	SARS-CoV 2 Spike Protein bound to LY-CoV555
7l56	Cryo-EM structure of the SARS-CoV-2 spike glycoprotein bound to Fab 2-43
7l57	Cryo-EM structure of the SARS-CoV-2 spike glycoprotein bound to Fab 2-15
7l58	Cryo-EM structure of the SARS-CoV-2 spike glycoprotein bound to Fab H4
7l5b	Crystallographic structure of neutralizing antibody 2-15 in complex with SARS-CoV-2 spike receptor-binding Domain (RBD).
7laa	Structure of SARS-CoV-2 S protein in complex with Receptor Binding Domain antibody DH1041
7lab	Structure of SARS-CoV-2 S protein in complex with N-terminal domain antibody DH1052

PDB ID	Description
7lcn	Structure of SARS-CoV-2 S protein in complex with N-terminal domain antibody DH1050.1
7ld1	Structure of SARS-CoV-2 S protein in complex with Receptor Binding Domain antibody DH1047
7lfz	Human leukocyte antigen B*07:02 in complex with SARS-CoV2 epitope IPRRNVATL
7lg0	Human leukocyte antigen B*07:02 in complex with SARS-CoV2 epitope SPRWYFYYL
7lg2	Human leukocyte antigen A*0201 in complex with SARS-CoV2 epitope ALWEIQQVV
7lg3	Human leukocyte antigen A*0201 in complex with SARS-CoV2 epitope KLWAQCVQL
7ljr	SARS-CoV-2 Spike Protein Trimer bound to DH1043 fab
7llf	SARS-CoV-2 papain-like protease (PLpro) bound to inhibitor XR8-83
7lm8	Crystal structure of SARS-CoV-2 spike protein receptor-binding domain in complex with two cross-neutralizing antibodies CV38-142 and COVA1-16 Fabs isolated from COVID-19 patients
7lop	Crystal structure of SARS-CoV-2 receptor binding domain in complex with antibodies CV05-163 and CR3022
7lqv	Cryo-EM structure of NTD-directed neutralizing antibody 4-8 Fab in complex with SARS-CoV-2 S2P spike
7lqw	Cryo-EM structure of NTD-directed neutralizing antibody 2-17 Fab in complex with SARS-CoV-2 S2P spike
7ls9	Cryo-EM structure of neutralizing antibody 1-57 in complex with prefusion SARS-CoV-2 spike glycoprotein
7lss	Cryo-EM structure of the SARS-CoV-2 spike glycoprotein bound to Fab 2-7
7lxw	SARS-CoV-2 S/S2M11/S2X333 Local Refinement

Supplementary Material

PDB ID	Description
7lxx	SARS-CoV-2 S/S2M11/S2L28 Local Refinement
7lxy	SARS-CoV-2 S/S2M11/S2X333 Global Refinement
7lxz	SARS-CoV-2 S/S2M11/S2L28 Global Refinement
7ly0	SARS-CoV-2 S/S2M11/S2M28 Local Refinement
7ly2	SARS-CoV-2 S/S2M11/S2M28 Global Refinement
7ly3	Crystal structure of SARS-CoV-2 S NTD bound to S2M28 Fab
7m3i	Structure of SARS-CoV-2 spike protein receptor binding domain in complex with a neutralizing antibody, CV2-75 Fab
7m6d	Structure of the SARS-CoV-2 RBD in complex with neutralizing antibodies BG4-25 and CR3022
7m6e	Structure of the SARS-CoV-2 S 6P trimer in complex with the human neutralizing antibody Fab fragment, BG10-19
7m6f	Structure of the SARS-CoV-2 S 6P trimer in complex with the human neutralizing antibody Fab fragment, BG1-22
7m6g	Structure of the SARS-CoV-2 S 6P trimer in complex with the human neutralizing antibody Fab fragment, BG7-15
7m6h	Structure of the SARS-CoV-2 S 2P trimer in complex with the human neutralizing antibody Fab fragment, BG7-20
7m6i	Structure of the SARS-CoV-2 S 2P trimer in complex with the human neutralizing antibody Fab fragment, BG1-24
7m7w	Antibodies to the SARS-CoV-2 receptor-binding domain that maximize breadth and resistance to viral escape
7mf1	Crystal structure of SARS-CoV-2 receptor binding domain in complex with neutralizing antibody 47D1

PDB ID	Description
7mmo	LY-CoV1404 neutralizing antibody against SARS-CoV-2
7nd3	EM structure of SARS-CoV-2 Spike glycoprotein in complex with COVOX-40 Fab
7nd4	EM structure of SARS-CoV-2 Spike glycoprotein in complex with COVOX-88 Fab
7nd5	EM structure of SARS-CoV-2 Spike glycoprotein in complex with COVOX-150 Fab
7nd6	EM structure of SARS-CoV-2 Spike glycoprotein in complex with COVOX-40 Fab
7nd7	EM structure of SARS-CoV-2 Spike glycoprotein in complex with COVOX-316 Fab
7nd8	EM structure of SARS-CoV-2 Spike glycoprotein in complex with COVOX-384 Fab
7nd9	EM structure of SARS-CoV-2 Spike glycoprotein (one RBD up) in complex with COVOX-253H55L Fab
7nda	EM structure of SARS-CoV-2 Spike glycoprotein (all RBD down) in complex with COVOX-253H55L Fab
7ndb	EM structure of SARS-CoV-2 Spike glycoprotein in complex with COVOX-253H165L Fab
7ndc	EM structure of SARS-CoV-2 Spike glycoprotein (all RBD down) in complex with COVOX-159
7ndd	EM structure of SARS-CoV-2 Spike glycoprotein (one RBD up) in complex with COVOX-159
7neg	Crystal structure of the N501Y mutant receptor binding domain of SARS-CoV-2 Spike glycoprotein in complex with COVOX-269 Fab
7neh	Crystal structure of the receptor binding domain of SARS-CoV-2 Spike glycoprotein in complex with COVOX-269 Fab
7ntc	Trimeric SARS-CoV-2 spike ectodomain bound to P008_056 Fab
7nx6	Crystal structure of the receptor binding domain of SARS-CoV-2 Spike glycoprotein in complex with COVOX-222 and EY6A Fabs

Supplementary Material

**PDB ID**	**Description**
7nx7	Crystal structure of the K417N mutant receptor binding domain of SARS-CoV-2 Spike glycoprotein in complex with COVOX-222 and EY6A Fabs
7nx8	Crystal structure of the K417T mutant receptor binding domain of SARS-CoV-2 Spike glycoprotein in complex with COVOX-222 and EY6A Fabs
7nx9	Crystal structure of the N501Y mutant receptor binding domain of SARS-CoV-2 Spike glycoprotein in complex with COVOX-222 and EY6A Fabs
7nxa	Crystal structure of the receptor binding domain of SARS-CoV-2 B.1.351 variant Spike glycoprotein in complex with COVOX-222 and EY6A Fabs
7nxb	Crystal structure of the receptor binding domain of SARS-CoV-2 P.1 variant Spike glycoprotein in complex with COVOX-222 and EY6A Fabs

Table S2. RMSD concerning the starting structure, packing and mobility parameters recovered at the respective MD simulations for the WT and P1 RBD.

Protein-Cluster	***%***	***RBD-RMSD[1*] (Å)***	***RBM-RMSD[2*] (Å)***	***Av. Non-polar Packing for Carbons in residue 417[3*]***	***Loop β1'/β2'-C RMSD (Å)[4*]***	***B1'-β2' Sheet RMSD (Å)[4*]***	***Loop B2'/η1' RMSD (Å)[4*]***	***Loop α1'/β1' RMSD (Å)[4*]***	***RBM "Claw" Rav (Å)[5*]***
**WT-C0**	89.6	1.306	0.716	1.5	1.699	1.471	1.878	1.677	25.96
**WT-C1**	8.4	1.359	1.696	0.5	2.287	1.743	2.512	1.812	26.56
**WT-C2**	1.9	1.797	1.101	1.5	1.890	1.567	2.104	1.764	25.96
WT-weight-Av.[6*]	-	*1.319*	*0.806*	*1.4*	*1.752*	*1.495*	*1.935*	*1.691*	*26.01*
WT-weighted fluctuation[7*]	-	$8.34 \cdot 10^{-2}$	$33.7 \cdot 10^{-2}$	$34.0 \cdot 10^{-2}$	$20.1 \cdot 10^{-2}$	$9.32 \cdot 10^{-2}$	$21.7 \cdot 10^{-2}$	$4.75 \cdot 10^{-2}$	$20.4 \cdot 10^{-2}$
**P1-C0**	98.1	1.196	1.021	2.0	1.791	1.476	2.103	2.603	26.12
**P1-C1**	1.5	1.111	1.397	1.0	2.742	2.419	2.274	2.760	26.72
**P1-C2**	0.3	1.074	0.989	0.5	2.818	2.097	1.885	2.162	26.34
P1-wheight-Av.[6*]	-	*1.194*	*1.027*	*2.0*	*1.836*	*1.491*	*2.105*	*2.604*	*26.13*
P1-weighted fluctuation[7*]	-	$1.50 \cdot 10^{-2}$	$5.61 \cdot 10^{-2}$	$18.1 \cdot 10^{-2}$	$16.1 \cdot 10^{-2}$	$14.6 \cdot 10^{-2}$	$2.94 \cdot 10^{-2}$	$3{,}78 \cdot 10^{-2}$	$9.05 \cdot 10^{-2}$

Supplementary Material

1* – RMSD considering de Cα atoms of the entire RBD from the cluster centroid structure related to the crystal (6M0J) or model (MODELLER) structure firstly taken to E-Volve Analysis.

2*- RMSD considering de Cα atoms of the entire RBM from the cluster centroid structure related to the crystal (6M0J) or model (MODELLER) structure firstly taken to E-Volve Analysis.

3* – Average non-polar packing calculated from the number of non-hydrogen atoms from hydrophobic residue side chains (Ala, Leu, Val,Ile, Pro, Phe, Met, Trp) around 4 Å from the also hydrophobic atoms (carbons and non-polar hydrogens) at the side chain from the residue at position 417 (Lys for WT; Thr for P1), divided by the number of carbons at this same side chain (respectively 4 for the Lys and 2 for the Thr).

4*- RMSD considering de Cα atoms from the RBM major elements involved in interactions with the receptor and/or antibodies . The C- terminal extension from Loop β1'/β2' (Loop β1'/β2'-C) – residues 470-490; β1'-β2' sheet – residues 451-455 (β1') and residues 492-496 (β2'); Loop β2'/η1' – residues 497-506; Loop α1'/β1' – residues 438-450. The alignment was carried out in relation to the RBM region.

5*- Average distance between the Cα atoms from the residues at the center of the three major loops defining the "claw" with high affinity to the receptor and to antibodies: Loop β1'/β2' (residue 480); Loop β2'/η1' (geometrical center from residues 501 and 502); Loop α1'/ β1' (residue 444).

6*- Weighted average of the measures considering the respective percentage of each cluster as the weights.

7*- Fluctuation calculated as the weighted standard deviation considering the respective percentages of each cluster as the weights and using the formula in Methods section. This estimation only considers the inter-cluster fluctuation of each measurement and not the internal fluctuations on each cluster (for the position related global fluctuations, the RMSF at Figure 5 A-B provides more accurate estimations).
